# Maternal and obstetric outcomes are influenced by developmental stage and cryopreservation of transferred embryos after clomiphene citrate-based minimal stimulation IVF

**DOI:** 10.1093/hropen/hoac018

**Published:** 2022-04-08

**Authors:** Sachie Onogi, Kenji Ezoe, Nami Kawasaki, Hiroko Hayashi, Tomoko Kuroda, Kazumi Takeshima, Kaou Tanoue, Shogo Nishii, Keiichi Kato

**Affiliations:** Kato Ladies Clinic, Shinjuku-ku, Tokyo, Japan; Kato Ladies Clinic, Shinjuku-ku, Tokyo, Japan; Kato Ladies Clinic, Shinjuku-ku, Tokyo, Japan; Kato Ladies Clinic, Shinjuku-ku, Tokyo, Japan; Kato Ladies Clinic, Shinjuku-ku, Tokyo, Japan; Kato Ladies Clinic, Shinjuku-ku, Tokyo, Japan; Kato Ladies Clinic, Shinjuku-ku, Tokyo, Japan; Kato Ladies Clinic, Shinjuku-ku, Tokyo, Japan; Kato Ladies Clinic, Shinjuku-ku, Tokyo, Japan

**Keywords:** clomiphene citrate, congenital anomalies, fresh embryo transfer, frozen embryo transfer, hypertensive disorders in pregnancy, low birthweight, low-lying placenta, placenta previa, pregnancy complications, preterm delivery

## Abstract

**STUDY QUESTION:**

Is the embryo transfer (ET) method associated with maternal and perinatal outcomes after minimal stimulation IVF using clomiphene citrate (CC)?

**SUMMARY ANSWER:**

The incidence of pregnancy complications and adverse perinatal outcomes was influenced by the developmental stage (cleavage versus blastocyst stages) and cryopreservation (fresh versus vitrified) of the transferred embryos.

**WHAT IS KNOWN ALREADY:**

Pregnancies resulting from IVF are associated with higher risks of adverse perinatal outcomes compared to natural conceptions; therefore, the next focus in reproductive medicine should be to assess whether these increased risks are attributable to IVF. Pregnancy complications and perinatal outcomes should be considered in addition to pregnancy outcomes when selecting the ET method, however, studies that describe the influence of transfer methods on perinatal and maternal outcomes are limited.

**STUDY DESIGN, SIZE, DURATION:**

This study retrospectively analysed a large single-centre cohort. The clinical records of 36 827 women who underwent oocyte retrieval (during a CC-based minimal stimulation cycle) followed by their first ET at the fertility treatment centre between January 2008 and December 2017 were retrospectively analysed. The patients underwent a single fresh cleavage-stage ET (SFCT), single vitrified-warmed cleavage-stage ET (SVCT) or single vitrified-warmed blastocyst transfer (SVBT). This study only included one cycle per patient.

**PARTICIPANTS/MATERIALS, SETTING, METHODS:**

Oocyte retrieval was performed following CC-based minimal ovarian stimulation. The embryos were transferred 2–3 days after retrieval or vitrified at the cleavage or blastocyst stage. The vitrified embryos were then warmed and transferred within the natural cycles. Pregnancy complications and perinatal outcomes were stratified according to the transfer methods used. Multivariate logistic regression analysis was performed to evaluate the effect of ET methods on the prevalence of pregnancy complications and congenital anomalies.

**MAIN RESULTS AND THE ROLE OF CHANCE:**

The rates of clinical pregnancy and delivery were significantly different among the groups. We analysed pregnancy complications in 7502 singleton births (SFCT, 3395 cycles; SVCT, 586 cycles; and SVBT, 3521 cycles). Multivariate logistic regression analysis revealed that the adjusted odds ratio (AOR) for hypertensive disorders in pregnancy was significantly lower in the SVBT group than in the SFCT group [AOR, 0.72; 95% CI, 0.56–0.92]. The AOR for low-lying placenta was lower in the SVBT group than in the SFCT group (AOR, 0.34; 95% CI, 0.19–0.60). The AOR for placenta previa was lower in the SVCT and SVBT groups than in the SFCT group (AOR, 0.21; 95% CI, 0.07–0.58 versus AOR, 0.53; 95% CI, 0.38–0.75, respectively). A total of 7460 follow-up data on neonatal outcomes was analysed. The AOR for preterm delivery was lower in the SVBT group than in the SFCT group (AOR, 0.78; 95% CI, 0.64–0.94). The AOR for low birthweight was significantly lower after SVCT and SVBT than after SFCT (AOR, 0.68; 95% CI, 0.46–0.98 versus AOR, 0.57; 95% CI, 0.48–0.66, respectively). The AOR for small for gestational age was lower in the SVCT and SVBT groups than in the SFCT group (AOR, 0.68; 95% CI, 0.46–0.98 versus AOR, 0.44; 95% CI, 0.36–0.55, respectively). The AOR for large for gestational age babies was higher in the SVBT group than in the SFCT group (AOR, 1.88; 95% CI, 1.62–2.18). The incidence of each congenital anomaly was similar among the groups.

**LIMITATIONS, REASONS FOR CAUTION:**

The study data were collected through self-reported parental questionnaires on maternal and neonatal outcomes. Our findings were not compared with the incidence of pregnancy complications and congenital anomalies in natural pregnancies. Furthermore, this study was retrospective in nature; therefore, further studies are required to ascertain the generalizability of these findings to other clinics with different protocols and/or different patient demographics.

**WIDER IMPLICATIONS OF THE FINDINGS:**

This study demonstrated reassuring outcomes for SVBT (in terms of a lower incidence of pregnancy complications) compared to SFCT. Our findings provide valuable knowledge that will help improve perinatal and maternal outcomes in CC-based stimulation and inform couples of the possible benefits and risks of each type of ET method.

**STUDY FUNDING/COMPETING INTEREST(S):**

This research did not receive any specific grants from funding agencies in the public, commercial, or not-for-profit sectors. The authors have no conflicts of interest to declare.

**TRIAL REGISTRATION NUMBER:**

N/A.

WHAT DOES THIS MEAN FOR PATIENTS? Pregnancies resulting from IVF treatment have higher risks (related to health of the mother and child) compared to natural conceptions. Therefore, effectiveness (pregnancy outcomes) and safety (maternal and child health) should be considered by clinicians when choosing treatment strategies, such as embryo transfer (ET) methods. The ET methods can be roughly divided into two types: fresh ET and frozen ET (FET). The outcomes of fresh ETs can be adversely affected by the medicine for egg growth promotion. By using FET, these adverse effects can be avoided; however, the embryos might be damaged by the cryopreservation (freezing) procedure. In this study, we investigated whether there is a link between ET method and the safety of mothers and babies during pregnancy and delivery. The study reviewed 10 years’ worth of data from a single infertility centre and found that FET in the natural cycle had a higher chance of ending in a live birth and a lower risk of pregnancy complications compared to fresh ETs in the egg retrieval cycle. These findings provide valuable information that will help improve clinical outcomes and maternal and child health and will also help couples when considering the possible benefits and risks of each type of ET method.

## Introduction

Fresh cleavage-stage embryo transfer (ET) may be advantageous to embryonic development since the uterus can maintain the homeostasis of the embryo’s environment more effectively than *in vitro* conditions (Fernandez-Shaw *et al.*, 2015; Glujovsky *et al.*, 2016). However, the pregnancy rate is higher after blastocyst transfer than after cleavage-stage ET. Furthermore, cryopreservation techniques enable embryos to avoid the detrimental effects that ovarian stimulation exerts on endometrial function (Ubaldi *et al.*, 1997; Kolibianakis *et al.*, 2002; Shapiro *et al.*, 2011). Therefore, an optimal and patient-friendly strategy for ET (i.e. cleavage stage versus blastocyst stage or fresh versus cryopreserved) should be selected for individual patients to obtain better pregnancy outcomes.

Pregnancies resulting from IVF (multiple or single) are associated with a higher risk of adverse perinatal outcomes, such as low birthweight (LBW), small for gestational age (SGA) (Qin *et al.*, 2017), pre-eclampsia and placental anomalies (Romundstad *et al.*, 2006; Vermey *et al.*, 2019), compared to pregnancies that were conceived naturally. Therefore, the next focus in reproductive medicine should be to assess whether the increased risks are attributable to ART. Hence, in selecting ET methods, perinatal and maternal outcomes should be considered in addition to pregnancy outcomes. However, studies describing the influence of transfer methods on perinatal and maternal outcomes are limited. An earlier study reported that neonatal health parameters, including the prevalence of congenital malformations, after blastocyst transfers are similar to or slightly better than those after cleavage-stage ETs (Ishihara *et al.*, 2014). Furthermore, a cumulative meta-analysis study reported that pregnancies resulting from frozen ETs (FET) have relatively lower risks of preterm delivery (PTD), LBW and SGA babies compared to those resulting from fresh ETs, which have an increased risk of hypertensive disorders of pregnancy (HDP and large for gestational age (LGA) babies) (Maheshwari *et al.*, 2018). However, these studies included several methods of ovarian stimulation. Ovarian stimulation protocols may adversely affect perinatal outcomes, such as LBW (Santos *et al.*, 2010); therefore, the influence of transfer methods on perinatal and maternal outcomes should be evaluated in patients who received treatment under the same protocol. That is, the ovarian stimulation method in the oocyte retrieval cycles should be uniform to unify the effect of ovarian stimulation on embryos. Furthermore, studies on perinatal and maternal outcomes in clomiphene citrate (CC)-based minimal stimulation cycles are limited. Therefore, we analysed retrospectively a large single-centre cohort stratified by the ET method they received and assessed the perinatal and maternal outcomes after CC-based minimal ovarian stimulation for IVF.

## Materials and methods

### Study patients

In this study, women were included when their first ET was determined. All clinical records of women who underwent oocyte retrieval (during a CC-based minimal stimulation cycle) followed by their first ET at the Kato Ladies Clinic between January 2008 and December 2017 were analysed retrospectively (Supplementary Fig. S1). The patients underwent a single fresh cleavage-stage ET (SFCT), single vitrified-warmed cleavage-stage ET (SVCT) or single vitrified-warmed blastocyst transfer (SVBT). This study included only one cycle per patient. Only the data of patients with singleton pregnancies were included. The follow-up data from all patients who delivered were used for the analysis of pregnancy complications. Data on the completed follow-up on neonatal outcomes were compared among the three ET methods. Data of patients who had cervical incompetence were excluded from the analysis of neonatal outcomes. We classified infertility as ovulation (irregular menstruation caused by polycystic ovary syndrome or diminished ovarian reserve), tubal factor (diagnosed by hysterosalpingography), endometrial factor (diagnosed by hysteroscope), male factor (diagnosed by semen test), combined and unexplained (patients not diagnosed with any cause).

### Ethical approval

This retrospective cohort study was approved by the Institutional Review Board of Kato Ladies Clinic (approval number: 21-14). Written informed consent for the analysis of embryonic, pregnancy, maternal and perinatal outcomes was obtained from all patients at the time of first consultation.

### Minimal ovarian stimulation cycle IVF

The detailed protocol for minimal stimulation with CC has been previously reported (Kato *et al.*, 2018; Karakida *et al.*, 2020; Nishihara *et al.*, 2020). In brief, CC (50–100 mg/day; Fuji Pharma Co., Ltd., Tokyo, Japan) was administered orally (with an extended regimen) from the third day of the retrieval cycle to the day before induction of final oocyte maturation. Ovulation was triggered using a nasal spray containing the GnRH agonist, buserelin (Suprecur; Mochida Pharmaceutical Co., Ltd., Tokyo, Japan or Buserecur; Fuji Pharma Co., Ltd.).

Oocyte retrieval was performed 30–36 h after triggering, using a 21-G needle (Kitazato Corporation, Shizuoka, Japan) without anaesthesia or follicular flushing. Cumulus–oocyte complexes were collected, washed and transferred to human tubal fluid medium (Kitazato Corporation) with paraffin oil at 5% CO_2_ in air at 37°C for culture, until either conventional IVF was performed 3 h later (Ezoe *et al.*, 2019) or, in cases of ICSI, denudation was performed 4 h after oocyte retrieval (Ohata *et al.*, 2019; Ezoe *et al.*, 2020). All embryos were cultured at 37°C (gas phase: 5% O_2_, 5% CO_2_ and 90% N_2_) with 100% humidity in a water jacket or with non-humidified incubators (Astec Co. Ltd, Fukuoka, Japan). Embryo vitrification and warming were performed using Cryotop (Kitazato Corporation), as previously described (Mori *et al.*, 2015).

### Embryo transfer

The ET method to be used was determined after consultations with patients at the initiation of oocyte retrieval cycles. In our clinic, SFCT was basically proposed for the first ET to simplify the first treatment cycle. However, the freeze-all strategy was chosen if a CC-induced thin endometrium was observed. In cases where an endometrial polyp was observed during the oocyte retrieval cycle or where the day chosen for the transfer was inconvenient for the patient, the freeze-all strategy was also chosen. Furthermore, in some cases, SVBT was chosen for the first ET owing to issues with the fallopian tubes and previous ectopic pregnancies. The patient’s preferences were also considered. SFCTs and SVBTs were performed as previously described (Kato *et al.*, 2012; Nishihara *et al.*, 2020; Onogi *et al.*, 2020). ET was performed under vaginal ultrasound guidance using a specially designed soft silicone inner catheter (Kitazato Corporation); a single embryo was placed in a minimal volume in the upper part of the uterine cavity. In SFCT cycles, the cleavage-stage embryo was transferred on Day 2 or 3 after oocyte retrieval in the CC-based minimal stimulation cycle. In SVCT and SVBT cycles, the cleavage-stage embryo or blastocyst was transferred on Day 2 or 5, respectively, after ovulation in a natural cycle. Oral dydrogesterone (30 mg/day; Mylan EPD G.K., Tokyo, Japan) was administered routinely during the early luteal phase after the transfers. Maternal and neonatal outcomes were obtained from the questionnaire filled by patients after the infant’s 1-month examination. All pregnant women were invited to respond to the questionnaire at 9 weeks of gestation, in the second trimester, and after delivery. If they did not respond, we contacted them and asked about their outcomes.

### Study outcomes

The primary outcomes were maternal and obstetric outcomes and major congenital anomalies. Maternal and obstetric outcomes included HDP, gestational diabetes mellitus, haemolysis-elevated liver enzymes-low platelet count syndrome, preterm premature rupture of membrane, low-lying placenta, placenta previa, placenta accreta, placental abruption and caesarean section, while neonatal outcomes included gestational age [≤27 weeks, 28–31 weeks, 32–36 weeks, 37–41 weeks and ≥42 weeks], birthweight [<1000 g, 1000–1499 g, 1500–2499 g and ≥2500 g], SGA and LGA. When the edge of the placenta was <20 mm from the cervix but not overlying it, it was classified as a low-lying placenta. When the placenta completely covered the cervix, it was classified as placenta praevia (Jansen *et al.*, 2020).

The questionnaire requested information on the following: date and mode of delivery, sex, birthweight, length of the newborn(s), presence of any birth defect or anomaly and pregnancy complications. A live birth was defined as any delivery at ≥22 weeks of gestation. PTD was defined as delivery occurring at <37 weeks of gestation. LBW and very LBW were defined as birthweights of <2500 g and <1500 g, respectively. Perinatal mortality was defined as the sum of stillbirths (≥22 pregnancy weeks) and early (within 7 days) neonatal deaths. SGA and LGA were defined as birthweights below the 10th percentile and above the 90th percentile, respectively, according to the Japanese national reference for neonates (Itabashi *et al.*, 2010). Neonatal outcomes were obtained from questionnaires completed by mothers after the 1-month infant examination. Congenital anomalies were classified using the Q-codes of the International Statistical Classification of Diseases and Related Health Problems, 10th Revision, by reformatting the answers provided by the parents in the questionnaires (World Health Organization, 2016). Major congenital anomalies were classified according to the European Surveillance of Congenital Anomalies (EUROCAT) guidelines into the following 13 classes: nervous system; eyes; ears, face and neck; congenital heart defects; respiratory; oro-facial clefts; digestive system; abdominal wall defects; urinary; genital; limb; other anomalies/syndromes; and chromosomal. According to the EUROCAT guidelines revised in November 2021, the following cases were not registered: cases of cerebral palsy; and cases with only minor defects, excluding those associated with major anomalies.

### Statistical analyses

All statistical analyses were performed using JMP software (SAS, Cary, NC, USA). Proportion data were analysed using the Chi-squared test, while continuous parameters were compared using the one-way ANOVA, with significance determined using Tukey’s test for *post*  *hoc* analysis. Univariate logistic regression analysis was used to identify confounders that were potentially associated with maternal and perinatal outcomes. Furthermore, the association of the confounders with the ET method groups was analysed. Multivariate logistic regression analysis for the maternal and perinatal outcomes was used to adjust for bias (using the confounders) and verify the statistical significance (using Wald statistic). The parameters associated with maternal and perinatal outcomes or with the ET method groups were used as confounders. For the analysis of pregnancy complications, maternal age, BMI, smoking, previous delivery and cause of infertility were used as confounders. For the analysis of perinatal outcomes, maternal age, BMI, smoking, previous delivery, cause of infertility and infant sex were used as confounders. Odds ratios (ORs) and adjusted ORs (AORs) are reported with 95% CIs for each group. The SFCT group was used as the reference for logistic regression analysis. A *P*-value of <0.05 was considered statistically significant.

## Results

### Pregnancy complications after SFCT, SVCT and SVBT

A total of 36 827 ETs (SFCT, 23 738 cycles; SVCT, 3395 cycles; and SVBT, 9694 cycles) were performed during the study period (Table I). The rates of clinical pregnancy and delivery were significantly different among the groups. The follow-up data of 7502 patients who delivered were stratified according to the transfer methods used (Table II). The incidence of pregnancy complications was higher in the SFCT group than in the SVCT and SVBT groups. In particular, the incidence of HDP was higher in the SFCT group compared with the SVBT group. Furthermore, low-lying placenta and placenta previa were more frequently observed in the SFCT group than in the SVCT and SVBT groups. The association between transfer methods and pregnancy complications adjusted for maternal age and BMI was assessed using multivariate logistic regression analysis (Table III). The AOR for HDP was significantly lower in the SVBT group than in the SFCT group. The AORs for low-lying placenta and placenta previa were lower in the SVCT and SVBT groups than in the SFCT group.

**Table I:** Characteristics of the study cohort undergoing minimal ovarian stimulation for their first embryo transfer cycle.

	SFCT	SVCT	SVBT	*P*-value
Embryo transfer cycles, n	23 738	3395	9694	
Maternal age, mean ± SEM*	38.2 ± 0.0^a^	39.0 ± 0.1^b^	37.6 ± 0.0^c^	<0.0001
BMI, mean ± SEM*	20.8 ± 0.0^a^	20.9 ± 0.0^b^	20.7 ± 0.0^c^	<0.0001
Smoking, n (%)**	563 (2.4)^a^	98 (2.9)^a^	438 (4.5)^b^	<0.0001
Previous delivery, n (%)**	2889 (12.2)^a^	452 (13.4)^a^	1815 (18.7)^b^	<0.0001
Cause of infertility				
Ovulation, n (%)**	169 (0.7)^a^	38 (1.1)^b^	86 (0.9)^a,b^	0.0219
Tubal factor, n (%)**	739 (3.1)^a^	19 (0.6)^b^	1193 (12.3)^c^	<0.0001
Endometrial factor, n (%)**	1067 (4.5)^a^	270 (7.9)^b^	600 (6.2)^c^	<0.0001
Male factor, n (%)**	1662 (7.0)	212 (6.3)	625 (6.4)	0.0791
Combined, n (%)**	546 (2.3)^a^	96 (2.8)^a^	488 (5.0)^b^	<0.0001
Unexplained, n (%)**	19 555 (82.4)^a^	2760 (81.3)^a^	6702 (69.1)^b^	<0.0001
Oestradiol on the day of maturation trigger (pg/ml)	696.1 ± 2.3^a^	305.5 ± 1.2^b^	306.6 ± 1.0^b^	<0.0001
Endometrial thickness (mm)*	9.8 ± 0.0^a^	9.8 ± 0.1^a^	10.4 ± 0.0^b^	<0.0001
Clinical pregnancy, n (%)**	5558 (23.4)^a^	949 (28.0)^b^	5323 (54.9)^c^	<0.0001
Singleton pregnancy, n (%)**	5535 (99.6)^a^	941 (99.2)^a,b^	5279 (99.2)^b^	0.0177
Deliveries, n (%)**	3395 (14.3)^a^	586 (17.3)^b^	3521 (36.3)^c^	<0.0001

Values are presented as mean ± SEM or n (%). SFCT, single fresh cleaved embryo transfer; SVBT, single vitrified-warmed blastocyst transfer; SVCT, single vitrified-warmed cleaved embryo transfer.

^a–c^ Different superscript letters indicate a significant difference at *P* < 0.05 (*Chi-squared test, **one-way ANOVA/Tukey’s test for *post hoc* analysis).

**Table II:** Pregnancy complications during the perinatal period, stratified according to embryo transfer method.

	SFCT	SVCT	SVBT	*P*-value
Deliveries, n	3395	586	3521	
Pregnancy complications, n (%)	435 (12.8)^a^	48 (8.2)^b^	352 (10.0)^b^	<0.0001
Hypertensive disorders of pregnancy, n (%)	166 (4.9)^a^	20 (3.4)^a,b^	122 (3.5)^b^	0.0079
Gestational diabetes mellitus, n (%)	88 (2.6)	15 (2.6)	99 (2.8)	0.8348
HELLP syndrome, n (%)	9 (0.3)	0 (0)	6 (0.2)	0.3588
Preterm premature rupture of membranes, n (%)	17 (0.5)	1 (0.2)	15 (0.4)	0.5293
Low-lying placenta, n (%)	48 (1.4)^a^	3 (0.5)^a,b^	20 (0.6)^b^	0.0007
Placenta previa, n (%)	99 (2.9)^a^	4 (0.7)^b^	58 (1.7)^b^	<0.0001
Placenta accreta, n (%)	2 (0.1)	1 (0.2)	3 (0.1)	0.6692
Placental abruption, n (%)	15 (0.4)	2 (0.3)	15 (0.4)	0.9423
Others, n (%)	10 (0.3)	1 (0.2)	18 (0.5)	0.2373

Values are presented as mean ± SEM or n (%). HELLP, haemolysis-elevated liver enzymes-low platelet count; SFCT, single fresh cleaved embryo transfer; SVBT, single vitrified-warmed blastocyst transfer; SVCT, single vitrified-warmed cleaved embryo transfer.

^a,b^ Different superscript letters indicate a significant difference at *P* < 0.05 (Chi-squared test).

**Table III:** Multivariate logistic regression analysis of pregnancy complications.

Adverse neonatal outcomes	Group	Odds ratio (95% CIs)	*P*-value	Adjusted odds ratio* (95% CI)	*P*-value
Hypertensive disorders of pregnancy	SVCT	0.69 (0.42–1.08)	0.1037	0.68 (0.42–1.09)	0.1136
	SVBT	0.68 (0.55–0.88)	0.0030	0.72 (0.56–0.92)	0.0088
Gestational diabetes mellitus	SVCT	0.97 (0.56–1.69)	0.9156	0.89 (0.50–1.57)	0.6812
	SVBT	1.08 (0.81–1.45)	0.5838	1.21 (0.90–1.66)	0.2892
HELLP syndrome	SVCT	–	–	–	–
	SVBT	0.64 (0.23–1.80)	0.3990	0.78 (0.28–2.23)	0.7123
Preterm premature rupture of membrane	SVCT	0.33 (0.04–2.51)	0.2871	0.33 (0.04–2.49)	0.2827
	SVBT	0.84 (0.42–1.70)	0.6431	1.04 (0.50–2.15)	0.9151
Low-lying placenta	SVCT	0.35 (0.11–1.13)	0.0809	0.34 (0.10–1.09)	0.0682
	SVBT	0.39 (0.23–0.67)	0.0006	0.34 (0.19–0.60)	0.0002
Placenta previa	SVCT	0.22 (0.08–0.61)	0.0036	0.21 (0.07–0.58)	0.0028
	SVBT	0.55 (0.40–0.77)	0.0005	0.53 (0.38–0.75)	0.0002
Placenta accreta	SVCT	2.85 (0.25–31.50)	0.3925	2.74 (0.24–31.34)	0.4183
	SVBT	1.44 (0.24–8.64)	0.6877	2.01 (0.31–13.38)	0.4719
Placental abruption	SVCT	0.75 (0.17–3.32)	0.7145	0.75 (0.17–3.29)	0.7023
	SVBT	0.96 (0.46–1.97)	0.9156	0.97 (0.46–2.04)	0.9340

Reference: single fresh cleaved embryo transfer group. *Adjusted for preconception characteristics (maternal age, BMI, smoking and cause of infertility).

HELLP, haemolysis-elevated liver enzymes-low platelet count; SVBT, single vitrified-warmed blastocyst transfer, SVCT, single vitrified-warmed cleaved embryo transfer.

A *P*-value of <0.05 was considered statistically significant (multivariate logistic regression analysis/Wald statistic).

### Neonatal outcomes after SFCT, SVCT and SVBT

We obtained the completed follow-up data on 7477 (99.7%) cases. Of these, patients with cervical incompetence were excluded from the analysis; hence, we analysed neonatal outcomes and congenital anomalies in 7460 singleton pregnancies (SFCT, 3385 cycles; SVCT, 575 cycles; and SVBT, 3500 cycles; Table IV, Supplementary Fig. S1). There was no statistical difference in the stillbirth rate among the three groups.

**Table IV:** Neonatal outcomes, stratified by embryo transfer method.

	SFCT	SVCT	SVBT	*P*-value
Patients with deliveries, n	3395	586	3521	
Completed follow-up data on neonatal outcomes, n (%)*	3389 (99.8)^a^	575 (98.1)^b^	3513 (99.8)^a^	<0.0001
Patients without cervical incompetence, n	3385	575	3500	
Live birth, n (%)*	3362 (99.3)	570 (99.1)	3489 (99.7)	0.0536
Stillbirth, n (%)*	23 (0.7)	5 (0.9)	11 (0.3)	0.0536
**Live birth**				
Caesarean section rate, n (%)*	1070 (31.8)	163 (28.6)	1165 (33.4)	0.0546
Gestational age, weeks, mean ± SEM**	39.0 ± 0.0^a^	39.2 ± 0.1^b^	39.0 ± 0.0^a,b^	0.0325
Gestational age, <28 weeks, n (%)*	13 (0.4)	0 (0)	17 (0.5)	0.2304
Gestational age, 28–31 weeks, n (%)*	37 (1.1)^a^	1 (0.2)^b^	18 (0.5)^b^	0.0050
Gestational age, 32–36 weeks, n (%)*	197 (5.9)	29 (5.1)	164 (4.7)	0.0975
Gestational age, 37–41 weeks, n (%)*	3104 (92.3)^a^	537 (94.2)^a,b^	3282 (94.1)^b^	0.0104
Gestational age, ≥42 weeks, n (%)*	11 (0.3)	3 (0.5)	8 (0.2)	0.4363
Birth length, cm, mean ± SEM**	48.6 ± 0.0^a^	48.8 ± 0.1^a,b^	49.1 ± 0.0^b^	<0.0001
Birthweight, g, mean ± SEM**	2922.0 ± 8.0^a^	2975.9 ± 16.3^a^	3035.3 ± 7.6^b^	<0.0001
Birthweight, <1000 g, n (%)*	27 (0.8)^a^	0 (0)^b^	17 (0.5)^a,b^	0.0372
Birthweight, 1000–1499 g, n (%)*	24 (0.7)	2 (0.4)	17 (0.5)	0.3524
Birthweight, 1500–2499 g, n (%)*	388 (11.5)^a^	53 (9.3)^a^	227 (6.5)^b^	<0.0001
Birthweight, ≥2500 g, n (%)*	2923 (86.9)^a^	515 (90.4)^a,b^	3228 (92.5)^b^	<0.0001
Small for gestational age, n (%)*	280 (8.4)^a^	33 (5.8)^b^	138 (4.0)^c^	<0.0001
Large for gestational age, n (%)*	335 (10.0)^a^	70 (12.3)^a^	586 (16.8)^b^	<0.0001
Infant sex				
Male, n (%)*	1651 (49.1)^a^	260 (45.6)^a^	1838 (52.7)^b^	0.0006
Female, n (%)*	1711 (50.9)^a^	310 (54.4)^a^	1651 (47.3)^b^	0.0006
Infant death, n (%)*	8 (0.2)	0 (0)	7 (0.2)	0.5044
Birth defect, n (%)*	134 (4.0)	17 (3.0)	111 (3.2)	0.1498
**Stillbirth**				
Birth defect, n (%)*	4 (17.4)	1 (20.0)	1 (9.1)	0.7836

Values are presented as mean ± SEM or n (%). SFCT, single fresh cleaved embryo transfer; SVBT, single vitrified-warmed blastocyst transfer; SVCT, single vitrified-warmed cleaved embryo transfer.

^a–c^ Different superscript letters indicate a significant difference at *P* < 0.05 (*Chi-squared test, **one-way ANOVA/Tukey’s test for *post hoc* analysis).

The rate of PTD was higher after SFCT than after SVCT and SVBT. The incidence of SGA was significantly higher in the SFCT group than in the SVCT and SVBT groups. Furthermore, the incidence of SGA was significantly higher in the SVCT group than in the SVBT group. The incidence of LGA was significantly higher in the SVBT group than in the SFCT and SVCT groups. The rates of infant death and birth defects were comparable among the groups. In the stillbirth cycles, the incidence of birth defect was also comparable among the three groups. Furthermore, multivariate logistic regression analysis demonstrated that the AOR for PTD was lower in the SVBT group that in the SFCT group (Table V). The AOR for LBW was significantly lower after SVCT and SVBT. A higher AOR for SGA and lower AOR for LGA were observed in the SFCT group compared to the SVCT and SVBT groups.

**Table V:** Multivariate logistic regression analysis of neonatal outcomes.

Adverse neonatal outcomes	Group	Odds ratio (95% CIs)	*P*-value	Adjusted odds ratio* (95% CI)	*P*-value
Stillbirth	SVCT	1.28 (0.48–3.38)	0.6159	1.36 (0.51–3.64)	0.5314
	SVBT	0.46 (0.22–0.94)	0.0350	0.53 (0.31–1.05)	0.0563
Caesarean section	SVCT	0.85 (0.70–1.04)	0.1246	0.83 (0.68–1.02)	0.0784
	SVBT	1.07 (0.97–1.18)	0.1674	1.10 (0.99–1.22)	0.0773
Preterm delivery (<37 weeks)	SVCT	0.70 (0.47–1.03)	0.0736	0.76 (0.52–1.09)	0.1375
	SVBT	0.76 (0.62–0.92)	0.0060	0.78 (0.64–0.94)	0.0104
Low birthweight (<2500 g)	SVCT	0.71 (0.52–0.95)	0.0238	0.68 (0.50–0.91)	0.0101
	SVBT	0.53 (0.45–0.63)	<0.0001	0.57 (0.48–0.66)	<0.0001
Small for gestational age	SVCT	0.67 (0.46–0.97)	0.0382	0.68 (0.46–0.98)	0.0436
	SVBT	0.45 (0.36–0.55)	<0.0001	0.44 (0.36–0.55)	<0.0001
Large for gestational age	SVCT	1.26 (0.95–1.66)	0.0969	1.25 (0.95–1.66)	0.1143
	SVBT	1.82 (1.57–2.10)	<0.0001	1.88 (1.62–2.18)	<0.0001
Infant death	SVCT	–	–	–	–
	SVBT	0.84 (0.30–2.32)	0.7414	0.87 (0.31–2.44)	0.8009
Birth defect	SVCT	0.70 (0.42–1.17)	0.1754	0.72 (0.44–1.18)	0.1912
	SVBT	0.81 (0.63–1.04)	0.1034	0.84 (0.65–1.08)	0.1766

Reference: single fresh cleaved embryo transfer group. *Adjusted for maternal age and preconception characteristics (maternal age, BMI, smoking, previous delivery, cause of infertility and infant sex).

SVBT, single vitrified-warmed blastocyst transfer; SVCT, single vitrified-warmed cleaved embryo transfer.

A *P*-value of <0.05 was considered statistically significant (multivariate logistic regression analysis/Wald statistic).

### Detailed analysis of congenital anomalies

Congenital anomalies were categorized into 13 classes (Table VI and Supplementary Table SI). The incidence of each congenital anomaly was similar among the groups in the live-birth cycles. The most frequent congenital anomaly was congenital heart defects in the live-birth cycles in all groups. In the stillbirth cycles, the incidence of each congenital anomaly was similar among the groups, and chromosomal anomalies were observed in all groups.

**Table VI:** Congenital anomalies, stratified by embryo transfer method.

	SFCT	SVCT	SVBT	*P*-value
Live birth, n	3362	570	3489	
Nerve system, n (%)	11 (0.3)	0 (0)	6 (0.2)	0.1996
Eyes, n (%)	3 (0.1)	0 (0)	1 (0.0)	0.4726
Ears, face and neck, n (%)	3 (0.1)	0 (0)	2 (0.1)	0.7135
Congenital heart defects, n (%)	47 (1.4)	8 (1.4)	40 (1.2)	0.6277
Respiratory, n (%)	3 (0.1)	0 (0)	6 (0.2)	0.4237
Oro-facial clefts, n (%)	7 (0.2)	1 (0.2)	4 (0.1)	0.6264
Digestive systems, n (%)	9 (0.3)	1 (0.2)	8 (0.2)	0.8961
Abdominal defects, n (%)	2 (0.1)	1 (0.2)	0 (0)	0.1174
Urinary, n (%)	11 (0.3)	0 (0)	14 (0.4)	0.3062
Genital, n (%)	13 (0.4)	0 (0)	6 (0.2)	0.0965
Limb, n (%)	10 (0.3)	1 (0.2)	11 (0.3)	0.8503
Other congenital abnormalities, n (%)	16 (0.5)	1 (0.2)	14 (0.4)	0.5768
Chromosomal, n (%)	13 (0.4)	6 (1.1)	14 (0.4)	0.0757
Stillbirth, n	23	5	11	
Nerve system, n (%)	2 (8.7)	0 (0)	0 (0)	0.4803
Urinary, n (%)	1 (4.4)	0 (0)	0 (0)	0.6998
Chromosomal, n (%)	1 (4.4)	1 (20.0)	1 (9.1)	0.4821

Values are presented as n (%). SFCT, single fresh cleaved embryo transfer; SVBT, single vitrified-warmed blastocyst transfer; SVCT, single vitrified-warmed cleaved embryo transfer.

A *P*-value of <0.05 was considered statistically significant (Chi-squared test).

## Discussion

In this large retrospective cohort study of 36 827 ET cycles, we confirmed that the clinical pregnancy and delivery rates were highest after SVBT, followed by SVCT, and then SFCT. Our results also showed that pregnancies resulting from SFCT had a significantly higher incidence of HDP and PTD compared to those resulting from SVBT. Furthermore, the incidences of placenta previa, low-lying placenta, LBW and SGA were significantly higher after SFCT than after SVCT and SVBT, while the LGA rate was significantly lower in the SFCT group. The rates of stillbirth, infant death and birth defect were comparable among all three groups.

This study demonstrated the favourable outcomes of SVBT—improved pregnancy, maternal and perinatal outcomes. In contrast, compared to SVCT and SVBT, SFCT was associated with a higher risk of pregnancy complications, which might be linked to the type of ET (cleavage versus blastocyst or fresh versus frozen) or the endometrial preparation (ovarian stimulation cycle or natural cycle). FET is associated with improved neonatal outcomes with respect to the rates of PTD, LBW and SGA but has a higher risk of LGA compared to fresh ETs although their congenital anomaly rates are comparable (Kato *et al.*, 2012; Maheshwari *et al.*, 2018); this is consistent with our results. A number of studies reported that FET was associated with a higher risk of HDP (Imudia *et al.*, 2013; Liu *et al.*, 2013; Ishihara *et al.*, 2014; Opdahl *et al.*, 2015; Maheshwari *et al.*, 2016); however, our results showed that fresh ET (SFCT) had a higher risk of HDP than frozen blastocyst transfer (SVBT). The abovementioned studies were limited because the cohorts and interventions (embryonic stage, freezing method, endometrial preparation protocol and criteria in replacement cycles) varied. Furthermore, most studies that described an increased risk of HDP after FET included hormone replacement (HR) as part of endometrial preparation (Imudia *et al.*, 2013; Liu *et al.*, 2013; Ishihara *et al.*, 2014). FET carried out in HR cycles is associated with a higher risk of HDP compared to natural cycles (Saito *et al.*, 2019; Moreno-Sepulveda *et al.*, 2021); therefore, we considered that the risk of HDP was associated more with the method of endometrial preparation than with the type of transfer.

Our results demonstrated that the incidences of placenta previa and low-lying placenta were significantly higher after fresh ET than after FET. A previous study reported that the risk of placental abnormalities, such as placenta previa, was lower after FET compared to fresh ET (Sazonova *et al.*, 2011); our study confirmed this result. However, some studies reported that the incidence of placental abnormalities was comparable between fresh ET and FET (Liu *et al.*, 2013; Ishihara *et al.*, 2014). One study showed that fresh ETs in the stimulated cycles and FET in the HR cycles were both associated with a higher risk of placental abnormalities compared to FET in natural cycles (Rombauts *et al.*, 2014). Thus, exogenous hormone administration for endometrial preparation might have a greater influence on the risk of placental abnormalities than the type of transfer.

Ovarian stimulation is associated with an increased risk of PTD, LBW and SGA compared to fresh ET in natural cycles (Jwa *et al.*, 2019). In the process of implantation and placentation, hormones stimulate trophoblast differentiation and invasion, which are essential during implantation (Malassine and Cronier, 2002; Pereira *et al.*, 2015). Recent studies have suggested that the supraphysiologic oestradiol milieu generated during fresh IVF could alter the optimal peri-implantation uterine environment, leading to abnormal placentation and ultimately, adverse perinatal and maternal outcomes, such as LBW, SGA, HDP and placenta previa (Bielefeldt *et al.*, 1990; Farhi *et al.*, 2010; Pereira *et al.*, 2015, 2017; Saito *et al.*, 2019; Huang *et al.*, 2020). When the serum oestradiol level on the day of the trigger is increased by ovarian stimulation, the increase in adverse obstetric outcomes continues to rise in a linear fashion (Royster *et al.*, 2016). In the present study, the serum oestradiol level on the day of maturation trigger was significantly higher in the SFCT group than in the SVCT and SVBT groups. Therefore, we hypothesized that a supra-physiological oestradiol level may alter normal angiogenesis and placentation, leading to adverse outcomes, such as LBW, SGA, HDP and placental abnormalities. Further investigations of potential alterations in placental and foetal development in pregnancies following fresh ET in a CC-based minimal stimulation cycle are needed.

### Strengths and limitations

The strength of our study is the large, single-centre cohort analysis since a large, uniform cohort is essential for the assessment of infrequent events such as minor obstetrical complications and congenital malformations. In the present study, all transferred embryos were derived from the oocytes retrieved in the CC-based minimal stimulation cycle, i.e. all embryos were exposed to a single treatment. This consistency helped us to exclude the effects of ovarian stimulation on the embryos. Furthermore, the laboratory and ET protocols and luteal support were uniform among the groups. In particular, FET was only performed in natural cycles, which simplified the comparison of the fresh and frozen transfer methods. Moreover, to overcome some of the inherent design limitations, we adjusted for important pregnancy-related confounders, such as maternal age and BMI, cause of infertility, smoking history and previous delivery, in our analyses to minimize possible flaws in our data. This improved the validity of our analysis, although unknown confounders and residual limitations may exist. First, the single-centre cohort analysis (which we mentioned as a strength above) could also be a limitation. The minimal stimulation protocol—extended administration of CC, intra-nasal GnRH agonist for ovulation trigger and oral progesterone for luteal support—is not widely used. Therefore, external validity of the study findings could be limited. Data were collected using self-reported parental questionnaires on maternal and neonatal outcomes. Self-reported maternal and neonatal complications could be potentially erroneous, particularly where uncommon/complex medical terms were involved. Furthermore, the collection rate of completed follow-up data on neonatal outcomes was 99.7%; some of the more severe outcomes could be associated with a lower chance of reply. Thus, a crosschecking process could have been more credible. Our findings were not compared with the incidence of pregnancy complications and congenital anomalies in natural pregnancy. Furthermore, this was a retrospective observational study, and there is a possibility that the difference in the patients’ background characteristics, which may have been caused by the indication of the ET methods, affected maternal and perinatal outcomes. Further studies comparing similar groups of patients are needed to ascertain the generalizability of these findings. Furthermore, we conducted power analysis on each outcome (deliveries, pregnancy complications and birth defects) among the ET methods and detected a difference of 99.9% for delivery, 99.9% for pregnancy complication and 92.2% for birth defects. However, this study showed powers ranging from 5.0% to 99.9% in detecting a difference in each complication or congenital anomaly among the groups; therefore, the accuracy of some analysis results was low owing to the small sample size. Therefore, further studies with larger sample sizes are required to validate our findings.

In conclusion, we found reassuring outcomes with SVBT in terms of lower incidence of pregnancy complications (including PTD, LBW, SGA, HDP) and placental abnormalities, compared to SFCT. Even with the growing trend to ‘freeze-all’ and an ‘elective FET’ policy, considering the higher risk of LGA it is premature to apply this policy to all ART cycles. Additionally, some couples opt for the fresh ET approach to simplify their first treatment cycle. It is important for practitioners to facilitate individualized treatment according to the clinical situation. Lastly, we mentioned the potential effect of endometrial preparation methods on the endometrium in either fresh ET or FET. Regarding the adverse effect of ovarian stimulation on normal placentation, improved protocols (e.g. regimens that utilize minimal stimulation) could help alleviate negative effects. Even though our SFCT group used the minimal stimulation with CC alone, we found some adverse perinatal outcomes. Further studies comparing pregnancy and neonatal complications after fresh transfers in natural and CC-based minimal stimulation cycles are needed to confirm and clarify the association between ovarian stimulation and perinatal and maternal outcomes. A few clinics have adopted the use of CC for minimal stimulation. Therefore, our findings provide valuable knowledge that will improve the clinical outcomes of CC-based stimulation. It is crucial for practitioners to evaluate, and inform couples of, the possible benefits and risks involved with each ART treatment process.

## Data availability

The datasets used and/or analysed during the present study are available from the corresponding author on reasonable request.

## Authors’ roles

S.O. contributed to the interpretation and writing. K.E. contributed to the study design, data collection, analysis, interpretation and writing. N.K., H.H. and T.K. contributed to the data collection and interpretation of the data. K.Tak., K.Tan. and S.N. contributed to revising the manuscript. K.K. contributed to the study design, interpretation and writing. All authors read and approved the final manuscript.

## Funding

This research did not receive any specific grants from funding agencies in the public, commercial, or not-for-profit sectors.

## Conflict of interest

The authors have no conflicts of interest to declare.

## Supplementary Material

hoac018_Supplemental_Table

hoac018_Supplementary_Figure_S1
