# Supplementary material for: Maternal and obstetric outcomes are influenced by developmental stage and cryopreservation of transferred embryos after clomiphene citrate-based minimal stimulation IVF
Source: Hum Reprod Open. 2022 Apr 8;2022(2):hoac018. doi: 10.1093/hropen/hoac018 (PMC9113344; doi:10.1093/hropen/hoac018)
Supplement: hoac018_Supplemental_Table [file hoac018_supplemental_table.docx]

Supplementary Table SI. P values of univariate logistic regression analysis between confounders and outcomes

|  | Previous delivery | Infant sex |
| --- | --- | --- |
| Pregnancy complications | *P* = 0.4415 | – |
| Stillbirth | *P* = 0.0349 | – |
| Caesarean section | *P* = 0.1827 | *P* = 0.1731 |
| Preterm delivery | *P* = 0.6744 | *P* = 0.1175 |
| Low birth weight | *P* = 0.9548 | *P* = 0.0001 |
| Small for gestational age | *P* = 0.9004 | *P* = 0.7845 |
| Large for gestational age | *P* = 0.0075 | *P* = 0.1259 |
| Infant death | *P* = 0.0618 | *P* = 0.8273 |
| Birth defect | *P* = 0.0002 | *P* = 0.8489 |

Supplementary Table SII. Details of congenital malformations

| Subgroups | Diagnosis |
| --- | --- |
| Nervous system | Hydrocephalus, Spina bifida, |
| Eye | Microphthalmia, Congenital cataract |
| Ear, face and neck, | Anotia |
| Congenital heart defects | Ventricular septal defect, Atrial septal defect, Tetralogy of Fallot, Tricuspid atresia and stenosis, Pulmonary valve stenosis, Pulmonary valve atresia, Aortic valve atresia/stenosis, Mitral valve anomalies, Patent ductus arteriosus as only CHD in term infants (>=37 weeks), |
| Respiratory | Cystic adenomatous malformation of lung, |
| Oro-facial clefts | Cleft lip with or without palate, Cleft palate |
| Digestive system | Duodenal atresia, Atresia or stenosis of other parts of small intestine, Ano-rectal atresia and stenosis, Hirschsprung Disease, Atresia of bile ducts, Diaphragmatic hernia |
| Abdominal wall defects | Omphalocele |
| Urinary | Multicyclic renal dysplasia, Congenital hydronephrosis |
| Genital | Hypospadias, |
| Limb | Club foot - talipes equinovarus, Hip dislocation, Polydactyly, Syndactyly |
| Other anomalies/ syndromes | Skeletal dysplasia, Craniosynostosis, Situs inversus, Congenital skin disorders, Genetic syndromes + microdeletions |
| Chromosomal | Down syndrome, Patau syndrome/trisomy 13, Edward syndrome/trisomy 18, Turner syndrome |
