# Supplementary figures and images for: Maternal and obstetric outcomes are influenced by developmental stage and cryopreservation of transferred embryos after clomiphene citrate-based minimal stimulation IVF
Source: Hum Reprod Open. 2022 Apr 8;2022(2):hoac018. doi: 10.1093/hropen/hoac018 (PMC9113344; doi:10.1093/hropen/hoac018)

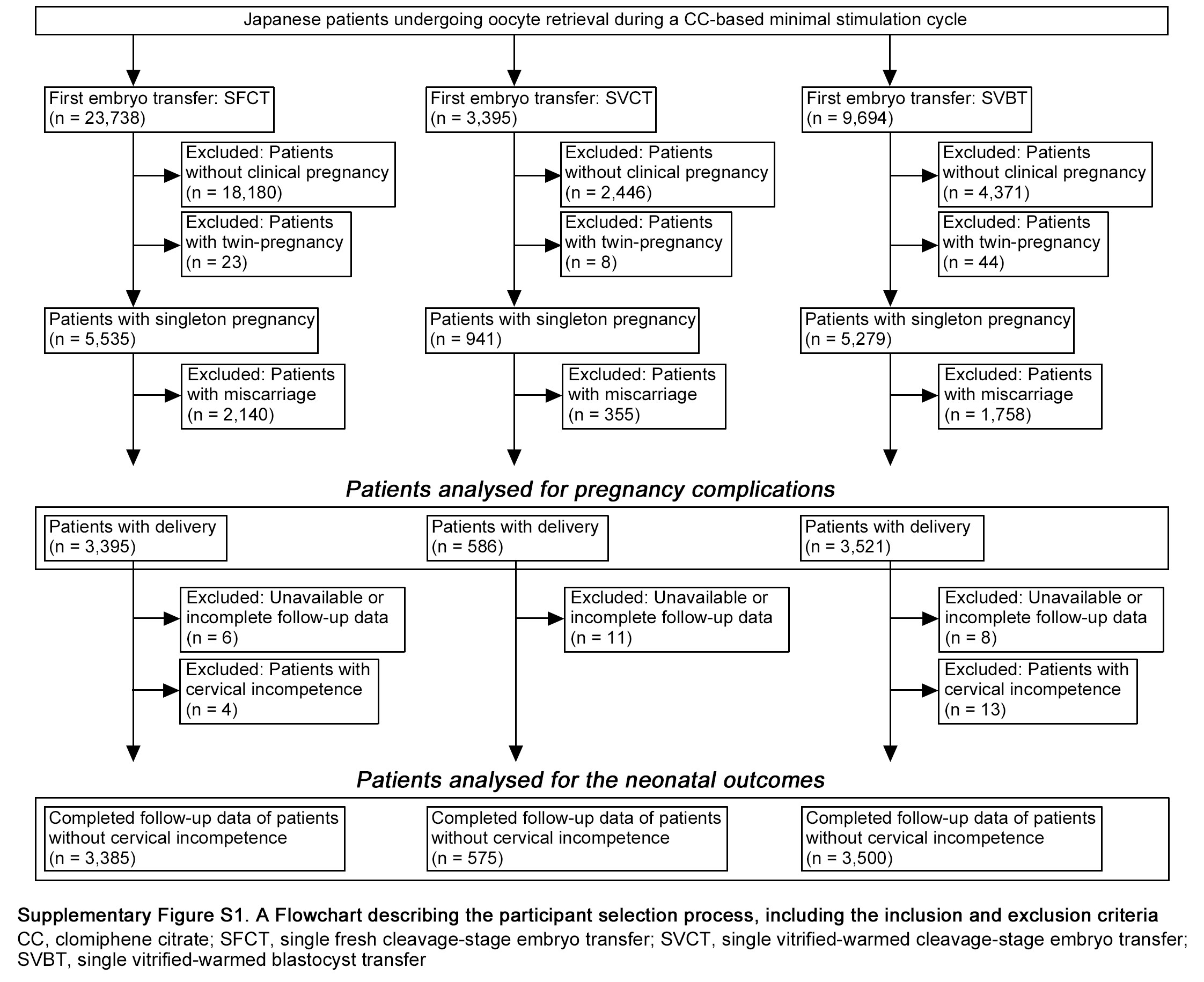

Supplement: hoac018_Supplementary_Figure_S1 [file hoac018_supplementary_figure_s1.jpeg]
